# Evaluating the effect of Neoadjuvant chemotherapy for esophageal Cancer using the RECIST system with shorter-axis measurements: a retrospective multicenter study

**DOI:** 10.1186/s12885-021-08747-y

**Published:** 2021-09-09

**Authors:** Yusuke Taniyama, Kentaro Murakami, Naoya Yoshida, Kozue Takahashi, Hisahiro Matsubara, Hideo Baba, Takashi Kamei

**Affiliations:** 1 Department of Surgery, Graduate School of Medicine, Tohoku University, 2-1 Seiryo-machi, Aoba-ku, Sendai, Miyagi 980-8575 Japan; 2 Department of Frontier Surgery, Graduate School of Medicine, Chiba University, Chiba, Japan; 3 Department of Gastroenterological Surgery, Graduate School of Medical Sciences, Kumamoto University, Kumamoto, Japan; 4 Department of Radiology, Graduate School of Medicine, Tohoku University, Sendai, Japan

**Keywords:** Esophageal neoplasms, Neoadjuvant therapy, Response evaluation criteria in solid tumors

## Abstract

**Background:**

Evaluating the effect on primary lesions is important in determining treatment strategies for esophageal cancer. The Response Evaluation Criteria in Solid Tumors system, which employs the longest diameter for measuring tumors, is commonly used for evaluating treatment effects. However, the usefulness of these criteria in assessing primary esophageal tumors remains controversial. Thus, we evaluated this issue by measuring not only the longest diameter but also the shorter axis of the tumor.

**Methods:**

We retrospectively reviewed data from 313 patients with esophageal cancer treated with neoadjuvant chemotherapy followed by esophagectomy at three major high-volume centers in Japan. All patients underwent contrast-enhanced computed tomography before and after chemotherapy. The longest and shortest tumor diameters were measured in each case. Treatment effects were adapted to the Response Evaluation Criteria in Solid Tumors system. Correlations between pathological and survival data were also analyzed.

**Results:**

Inter-observer discrepancies were examined for changes in the longest diameter and shorter axis of the tumor (the intraclass correlation coefficients were 0.550 and 0.624, respectively). The shorter axis was correlated with the pathological response in the multivariate analysis (*p* < 0.001). The shorter axis was significantly associated with overall survival and disease-free survival (both p < 0.001), whereas this association was not observed for the longest tumor diameter.

**Conclusions:**

This multicenter study demonstrated that the Response Evaluation Criteria in Solid Tumors system is useful for predicting pathological response and survival by incorporating the shorter axis of the primary esophageal tumor.

**Supplementary Information:**

The online version contains supplementary material available at 10.1186/s12885-021-08747-y.

## Background

Neoadjuvant therapies, such as neoadjuvant chemotherapy (NAC) and chemoradiotherapy, are well-established treatments for resectable esophageal cancer treatment [1–5]. Evaluating the treatment effect before surgery is important in determining the treatment strategy because the response to neoadjuvant therapy is a well-known predictor of survival [6]. Although reports have highlighted the role of positron emission tomography (PET) and other modalities for evaluating treatment effects in esophageal cancer [7–13], these modalities are not used in clinical practice due to their cost and complexity.

In contrast, computed tomography (CT) is commonly used in clinical settings for evaluating the response of esophageal cancer to treatment because it defines the local extent of the tumor, lymph node size, and existence of distant metastasis at relatively low cost while being reproducible and less invasive. The Response Evaluation Criteria in Solid Tumors (RECIST) (version 1.1) [14] system, which assesses the anatomical shrinkage of the tumor by measuring the longest tumor diameter, is widely used for evaluating treatment effects and can be applied to various types of tumors. However, primary esophageal tumors are occasionally classified as “non-measurable” because, unlike solid organ tumors, digestive organ tumors are usually not large or clear enough for appropriate measurement. Therefore, in the Japanese Classification of Esophageal Cancer (eleventh edition) [15], primary esophageal tumors have been defined as “non-measurable lesions.” This is problematic in advanced primary tumors without lymph node metastases because the effect of NAC cannot be evaluated accurately. To address this, the usefulness of the RECIST system in primary esophageal tumors must be understood. Some reports [10–12] suggested that the RECIST system does not reflect actual treatment effects; however, these studies had small sample sizes and employed only the longest tumor diameter for evaluation. Few reports have evaluated this issue in a sufficiently large cohort. The purpose of this study was to clarify the usefulness of the RECIST system adapted to the primary esophageal tumor.

## Methods

### Study design and patients

In this retrospective multicenter study, we aimed to evaluate the utility of the RECIST system adapted to primary esophageal tumors by measuring not only the longest tumor diameter but also the shortest tumor diameter. Data from patients with esophageal cancer treated with NAC followed by subtotal esophagectomy were retrospectively collected from three high-volume centers in Japan. These centers are located in different regions and provide medical care independently. Patients with incomplete medical records, including those without precise CT scans taken before and after NAC, were excluded. Additionally, we excluded 45 patients in whom the tumor could not be identified at least 1 cm along the esophageal wall using CT. Finally, 313 patients were enrolled (Tohoku University Hospital [January 2009 to December 2019], *n* = 81; Kumamoto University Hospital [August 2008 to April 2020], *n* = 149; and Chiba University Hospital [January 2001 to December 2019], *n* = 83). This study was approved by the appropriate ethics committees (approval numbers: 2020–1-114 [Tohoku], 2011 [Kumamoto], and 19,374 [Chiba]), and the need for written informed consent from the patients was waived.

### Treatment

Patients diagnosed with stage IB/II/III esophageal squamous cell carcinoma, according to the tumor-node-metastasis criteria, were administered NAC, as reported previously [1–5].

All patients underwent subtotal esophagectomy with radical lymphadenectomy through fifth intercostal thoracotomy or thoracoscopic procedures. The extent of lymphadenectomy was determined according to the Japan Esophageal Society (2012) guidelines [16]. Adjuvant treatment was considered for cases of non-curative resection. All specimens were pathologically diagnosed at each institutions’ Pathology Department. The Union for International Cancer Control (seventh edition) [17] was used for tumor-node-metastasis staging. Briefly, stage IB corresponds to T2 without lymph node metastasis; stage II corresponds to T1 or T2 with a few lymph node regional metastases; T3 is without lymph node metastasis; stage III is associated with T1–4 with regional lymph node metastases; and stage IV is associated with distant metastasis.

### Measurement of tumor size on CT scan

All patients underwent contrast-enhanced CT (arterial and venous phases) from the neck to the abdomen, with slices ranging from 0.5–5.0 mm. CT was performed before NAC and 2–4 weeks after, and esophagectomy was performed 4–6 weeks after the last administration of NAC. The tumor area was measured at a site with different contrast enhancement and thickness compared to those of the normal esophageal wall. Tumor diameters were measured using the horizontal slice at the thickest part of the tumor. The measurements of tumor diameter before and after NAC were performed using exactly the same horizontal slice and in the same direction. The longest diameter was measured as the maximum distance from the outer tumor margin. The shorter axis was measured on the same slice as the longest diameter and was defined as the maximum tumor size orthogonal to the longest diameter axis; this would represent tumor thickness. In most cases, the tumor lumen could be detected using the air in the esophageal lumen, feeding tube, or normal esophageal wall; when the lumen of the tumor could not be observed clearly, we considered half of the maximal cross-sectional diameter the shorter axis.

### Data evaluation

First, we examined the inter-examiner consistency of tumor size measurements by CT. We selected 81 cases (Tohoku University Hospital), and two experienced examiners (Y.T. and K.T.) measured the longest and shortest tumor diameters individually without access to the clinical data. Y.T. is a board-certified esophageal surgeon, and K.T. is a board-certified surgeon with 3 years of experience as a radiologist specializing in esophageal cancer. We then focused on the correlation between the measured treatment effect classified by the RECIST system and the clinical data, such as pathological and prognostic data. Data at each institution were measured by board-certified esophageal surgeons (Y.T., N.Y., and K.M.).

### Definition of treatment effect

The RECIST system was used to classify the treatment effects using CT. Although complete response (CR) is defined as tumor disappearance, we eliminated this category because it was impossible to determine tumor disappearance without performing a biopsy during endoscopic examination. Partial response (PR) was defined as a ≥ 30% decrease in the longest diameter and shorter axis. Progressive disease (PD) was defined as an increase of ≥20% in these diameters. Stable disease (SD) was defined as a disease that did not meet the criteria for PR/PD. Since these criteria were based only on changes in the primary lesion, the changes in metastatic lymph nodes or the occurrence of new lesions after NAC were not included in the analysis.

The tumor regression grade (TRG) [18], which has been proposed by the Japan Esophageal Society, was used to evaluate the treatment effect from a pathological perspective. All specimens were classified according to a 0–5 scale based on the proportion of residual cancer cells in the primary tumor. Subsequently, this assessment was reclassified into two groups: TRG 0–1a (“ineffective group,” which included patients in whom less than one-third of the tumor disappeared after treatment) and TRG 1b–3 (“effective group,” which included patients in whom more than one-third of the tumor disappeared).

### Statistical analyses

The intraclass correlation coefficient (ICC) was used to examine the consistency of tumor measurements between the two examiners. A receiver operating characteristic (ROC) curve was used to obtain the optimal diameter thresholds to determine the pathological response. Overall survival (OS) and disease-free survival (DFS) were analyzed using the Kaplan–Meier method and the log-rank test. OS was defined as the time from the date of surgery to the date of death from any cause. DFS was calculated from the date of surgery to the date of diagnosis of any cancer recurrence or death from any cause. Logistic regression analysis was used to assess the prediction of TRG from the treatment effect using the RECIST system. To evaluate the independent risk factors for OS according to preoperative factors and RECIST data, multivariate analysis was performed. Variables with *P* < 0.2 in the univariate analysis were entered into the multivariate analysis. Statistical analyses were performed using JMP Pro 15.0.0 statistical software (SAS Institute Inc., Cary, NC, USA). *P* < 0.05 was considered statistically significant.

## Results

The clinical and pathological characteristics of the patients are summarized in Table 1. Among the 313 patients, 87% were male, the mean age was 66.5 years, and 89% had squamous cell carcinoma, which is similar to the proportion of patients with esophageal cancer in Japan [1]. Platinum- and taxane-based regimens were administered as NAC in 203 (65%) and 110 (35%) patients, respectively. In the evaluation using the RECIST system, patients were distributed equally between the PR and SD groups based on the shorter axis measurements, whereas more patients were included in the SD group based on the longest diameter measurements. TRG 0–1a, which represented an insufficient treatment effect on pathological examination, was observed in almost two-thirds of the patients.

**Table 1:** Patient characteristics and clinical outcome (*n* = 313)

Clinical, epidemiological, pathological, and surgical features	Value
Mean age ± SD, years	66.5 ± 7.3
Male sex, n (%)	272 (87)
Performance status, n (%)	
0	222 (71)
1	88 (28)
2	3 (1)
Tumor location, n (%)	
Ce	1 (0.3)
Ut	42 (13)
Mt	144 (46)
Lt	98 (31)
Ae	28 (9)
Histopathological type, n (%)	
Squamous cell carcinoma	279 (89)
Adenocarcinoma	20 (6)
Others	14 (4)
Pretreatment clinical stage, n (%)	
IB	7 (2)
II	53 (17)
III	218 (70)
IV	35 (11)
NAC regimen, n (%)	
Platinum-based	203 (65)
Taxane-based	110 (35)
RECIST (longest diameter), n (%)	
PR	126 (40)
SD	174 (56)
PD	13 (4)
RECIST (shorter axis), n (%)	
PR	145 (46)
SD	150 (48)
PD	18 (6)
Severe morbidity, n (%)	
CDC ≥ IIIb	34 (11)
Pathological stage, n (%)	
0–I	60 (19)
II	88 (28)
III	158 (41)
IV	37 (12)
TRG	
0	16 (5)
1a	184 (59)
1b	49 (16)
2	45 (14)
3	19 (6)

*Abbreviations*: *Ae* abdominal esophagus, *CDC* Clavien–Dindo classification, *Ce* cervical esophagus, *Lt* lower thoracic, *Mt* mid-thoracic, *NAC* neoadjuvant chemotherapy, *PD* progressive disease, *PR* partial response, *RECIST* Response Evaluation Criteria in Solid Tumors, *SD* stable disease, *SD* standard deviation, *TRG* tumor regression grade, *Ut* upper thoracic

The examination of inter-observer discrepancies in tumor diameter measurements using CT revealed that the shorter axis was superior in evaluating changes in size (see Supplementary Table S1 in Additional File 1). The ICC value of the shorter axis was 0.624, which is good for objectivity and reproducibility and seems to be capable of evaluating the effect of NAC. In turn, the ICC value of the longest diameter was 0.550, which, although adequate, was not as good as that of the shorter axis.

The RECIST system showed a good correlation with the pathological treatment effect by logistic regression analysis (Table 2). In the multivariate analysis, changes in the shorter axis, rather than in the longest diameter or multiplication of both axes (tumor area), were an independent factor for TRG. This result was not affected by the tumor size (see Supplementary Table S2 in Additional File 1). Moreover, the shorter axis was associated with the pathological effect according to ROC curve analysis (area under the curve: 0.747) (see Supplementary Table S3 in Additional File 1). The optimal shorter axis reduction rate cut-off of 32.6% had a sensitivity of 67.2%, specificity of 71.0%, and accuracy of 64.5%. Additionally, three-dimensional measurement using the product of the shorter axis, longest diameter, and longitudinal distance showed results similar to those of the shorter axis.

**Table 2:** Logistic regression analysis for estimating pathologically ineffective response (TRG 0–1a)

Characteristic	Univariate analysis		Multivariate analysis	
	Odds ratio (95% CI)	*P*-value	Odds ratio (95% CI)	P-value
RECIST classification by longest diameter		< 0.001		0.888
PR (vs. SD + PD)	0.36 (0.23–0.59)		0.95 (0.43–2.32)	
RECIST classification by shorter axis		< 0.001		0.022
PR (vs. SD + PD)	0.24 (0.15–0.39)		0.43 (0.20–0.88)	
^a^Multiplication of longest diameter by shorter axis		< 0.001		0.112
PR (vs. SD + PD)	0.25 (0.15–0.40)		0.45 (0.17–1.21)	

^a^The cut-off value of PR and SD + PD in the multiplication of the longest diameter and the shorter axis was defined as 51%, which was calculated by multiplication of 30% reduction rate in both axes

*Abbreviations*: *CI* confidence interval, *PD* progressive disease, *PR* partial response, *RECIST* Response Evaluation Criteria in Solid Tumors, *SD* stable disease, *TRG* tumor regression grade

**Fig. 1:**
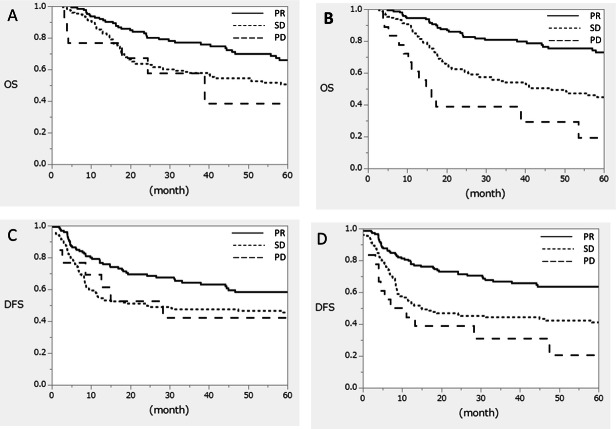
Kaplan–Meier curves after esophagectomy based on the effect of neoadjuvant chemotherapy. **A**, **B** Overall survival. **C**, **D** Disease-free survival. Patients were stratified based on the RECIST system, according to changes in the longest tumor diameter (**A**, **C**) and the shortest tumor diameter (**B**, **D**. RECIST, Response Evaluation Criteria in Solid Tumors; PR, partial response; SD, stable disease; PD, progressive disease; OS, overall survival; DFS, disease-free survival. Survival analyses are described in Table 3

Survival analyses are shown in Fig. 1 and Table 3. The mean follow-up for all patients and patients alive at the time of analysis were 1307 and 1661 days, respectively. Figure 1 shows the OS and DFS of the RECIST system classified by the longest diameter and shorter axis. The shorter axis was significantly associated with both OS and DFS, whereas the longest diameter was not. The results of univariate and multivariate analyses of OS are shown in Table 3. RECIST classification using the shorter axis was an independent prognostic factor for OS along with other factors such as TRG and pathological stage. Multiplication of the longest diameter and the shorter axis, which represents the tumor area, also correlated with OS in the univariate analysis. However, we did not include this parameter in the multivariate analysis because of its confounding effect on diameter and axis.

**Table 3:** Cox regression analysis of overall survival

Characteristic	Univariate analysis		Multivariate analysis	
	HR (95% CI)	P-value	HR (95% CI)	P-value
Age, years	1.15 (1.002–1.053)	0.037	1.06 (1.030–1.085)	< 0.001
Sex, male (vs. female)	1.41 (0.800–2.561)	0.232		
Performance status, 0 (vs. 1–2)	0.88 (0.593–1.313)	0.537		
NAC regimen, platinum-based (vs. taxane-based)	0.84 (0.591–1.194)	0.332		
RECIST classification by longest diameter; PR (vs. SD + PD)	0.68 (0.474–0.979)	0.038	1.15 (0.764–1.731)	0.501
RECIST classification by shorter axis; PR (vs. SD + PD)	0.40 (0.278–0.588)	< 0.001	0.56 (0.363–0.856)	0.008
RECIST classification by multiplication of longest diameter by shorter axis; PR (vs. SD + PD)	0.50 (0.344–0.720)	< 0.001	*Inappropriate for multivariate analysis*	
Histological type Squamous cell carcinoma (vs. others)	0.93 (0.512–1.683)	0.806		
TRG, ≥1b (vs. 0–1a)	0.26 (0.162–0.421)	< 0.001	0.35 (0.215–0.580)	< 0.001
Pathological stage 0–II (vs. III + IV)	0.31 (0.209–0.460)	< 0.001	0.32 (0.213–0.490)	< 0.001

*Abbreviation*s: *CDC* Clavien–Dindo classification, *CI* confidence interval; HR, hazard ratio, *NAC* neoadjuvant chemotherapy, *PD* progressive disease, *PR* partial response, *RECIST* Response Evaluation Criteria in Solid Tumors, *SD* stable disease, *TRG* tumor regression grade

## Discussion

This retrospective multicenter study showed that the RECIST system assessed using CT was useful for evaluating the effect of NAC on primary esophageal tumors. To allow widespread application, evaluations should be performed based on an imaging modality used in routine examinations. The advantage of using CT to evaluate the treatment effect is that this examination is necessary both before and after NAC. To determine the treatment strategy, clinicians use CT to assess tumor invasion and metastasis before starting treatment. At this point, PET is useful for detecting distant metastasis to avoid unnecessary invasive treatment. After NAC, CT is necessary for the surgeon to determine how the tumor has changed and plan surgery accordingly. However, PET is not necessary unless a new suspicious metastatic lesion is evident on CT imaging. Thus, CT is the desirable modality for evaluating the treatment effect of NAC on esophageal cancer.

**Fig. 2:**
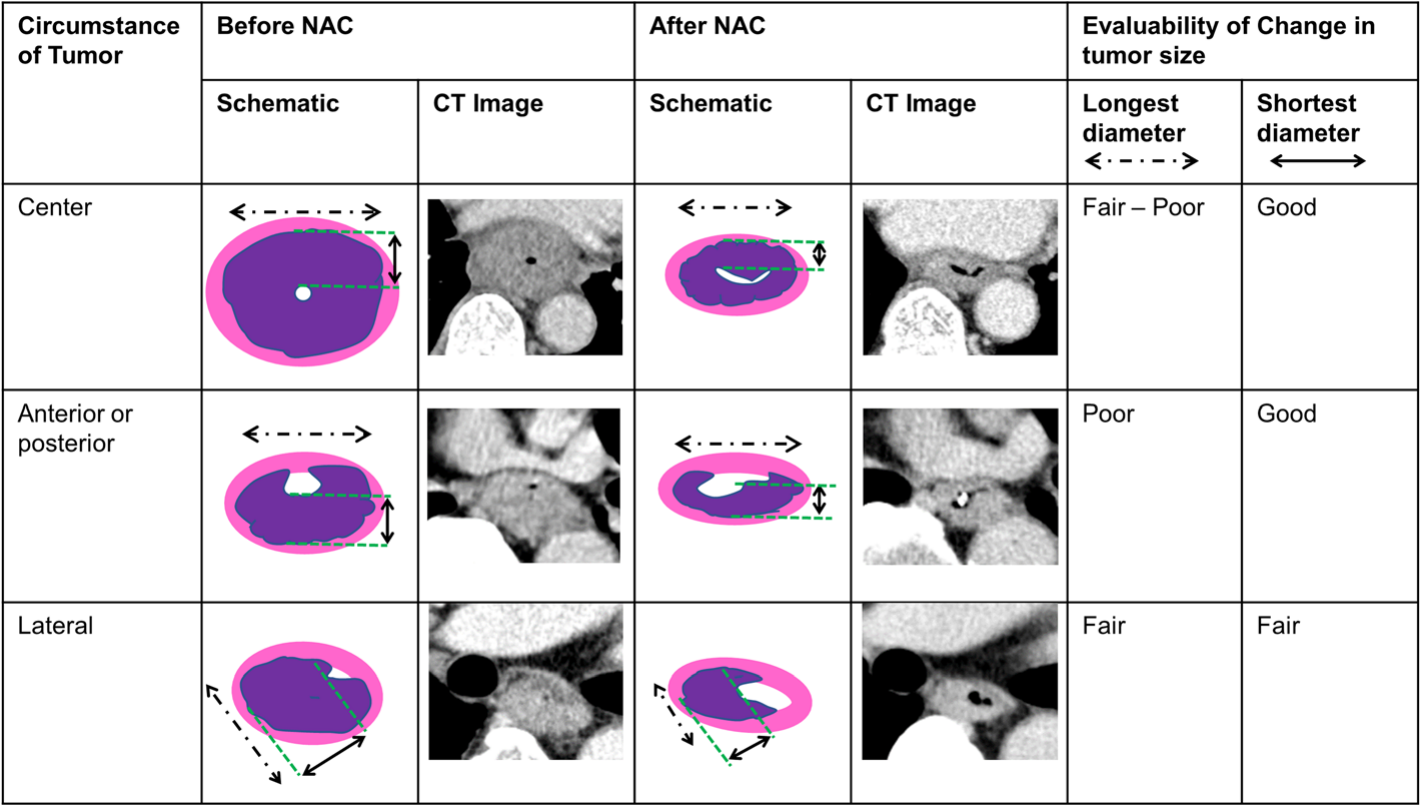
Measuring changes in tumor size using the shortest and longest diameters, depending on tumor location. The longest diameter is the maximum distance between the outer tumor margins (dotted arrow). The shorter axis is the distance between the inner and outer tumor margins; it represents the tumor thickness (solid arrow). Each measurement was performed in the same horizontal slice and the same direction before and after neoadjuvant chemotherapy. NAC, neoadjuvant chemotherapy; CT, computed tomography

Regardless of the imaging modality used, the evaluation method must also be simple enough to allow practical use (e.g., measuring the anatomical change in primary tumor size during routine examination). Although several methodologies that use CT have been studied to evaluate the treatment effect, they are too complicated to be adapted for clinical use [19–21]. The RECIST system is well known for evaluating the treatment effect, and many clinicians are familiar with it. Several reports [10–12] have used the longest diameter to evaluate the tumor by CT, the same as in the original RECIST system; however, these have not shown good results. Moreover, since the horizontal plane of the esophagus is originally an ellipse, it is often difficult to evaluate real changes in tumor size using the longest diameter (Fig. 2). For bulky tumors, the shorter axis appears to be the most reliable measure of changes in tumor size, although the longest diameter may also be adequate. However, in cases where the tumor is not so large and is located on one side of the esophagus, the longest diameter is inadequate. Thus, the shorter axis should be used to ease the evaluation of actual changes in tumor size.

A previous report [22] suggested that the anatomical tumor changes observed on CT could be the last phenomenon that occurs after NAC for esophageal cancer, compared to metabolic changes that are evident on PET. However, our results revealed that changes in tumor size are useful for determining the pathological response to NAC. Moreover, a 30% reduction rate in RECIST, defined as the borderline of PR and SD, seems appropriate when the shorter axis is adapted for tumor size measurement. Our ROC curve for pathological response showed that the most appropriate cut-off value was 32.6%, which was comparable to that in the RECIST system (Supplementary Table S3 in Additional File 1). These data also justify the use of the RECIST system with the shorter axis for primary esophageal tumors.

We also showed that changes in the shorter axis correlated with survival. The OS and DFS survival curves demonstrated superior outcomes in patients with PR according to the RECIST system using the shorter axis. Although the longest diameter did not demonstrate statistical significance, some trends in survival data were observed. This means that patients with apparent tumor shrinkage after NAC would have better survival, similar to the idea of pathological response. Based on these data, the measurement does not need to be performed strictly using the shorter axis; the shorter axis can be measured where the diameter appears to be the shortest. In addition, tumor area changes constitute another method for measuring the treatment effect. We evaluated this parameter by multiplying the longest diameter by the shorter axis and analyzed its correlation with the pathological effect and prognosis. Surprisingly, this multiplication parameter was inferior to the shorter axis in terms of measuring the treatment effect.

In this study, we did not use the CR criteria, an important phenomenon to be evaluated before surgery [8]. Although CT is advantageous for assessing anatomical changes and the relationships with surrounding organs, it is still impossible to detect a small number of cancer cells simply by monitoring tumor appearance. Among the 24 cases that showed tumor disappearance by CT, only four achieved pathological CR. Moreover, the four cases classified as TRG 1a did not demonstrate a sufficient response to NAC. Based on these data, pathological CR would be difficult to evaluate by CT. To evaluate the disappearance of cancer cells, biopsy during the endoscopic examination may be the best approach; however, this may still not be sufficient to determine pathological CR [23–25]. Currently, clinical CR does not reflect pathological CR, and surgical resection is still recommended in these cases [26, 27]. Therefore, CR evaluation may not be necessary for patients receiving NAC, or “near CR” should be established as a new criterion.

The disadvantage of CT is that there is some inter-examiner discrepancy compared to PET. Although our results showed that measurements of the shorter axis were relatively consistent between examiners, the ICC value was not ideal. Moreover, the issue regarding tumors that are too small to be detected by CT remains. Especially in the case of “near CR,” some small tumors were difficult to identify by venous phase of CT scan, which is the standard approach in the clinical setting. Unfortunately, there are no definitive answers to these questions yet, and perhaps other modalities, such as endoscopy or PET, should be combined in such cases. However, until these modalities are commonly used before and after NAC, measurement of the shorter axis by CT remains desirable for determining treatment efficacy.

Limitations of this study include its retrospective design and the risk of inter-institutional biases in CT devices and slice width; however, we believe that these biases had little effect on our results. Moreover, other inter-institutional biases, such as differences in NAC regimens, surgical procedures, perioperative management, and treatments after recurrence, might have been present. To verify our data, validation in other studies is needed. Additionally, the accurate measurement of esophageal lesions could be quite challenging in tumors with a distorted shape. If the tumor boundary is too indistinct to be measured, it may be better to use another method; this is currently under analysis by our multicenter group.

## Conclusions

This is the first multicenter study to evaluate the effect of NAC on esophageal cancer by adapting the RECIST system to assess the primary tumor. Changes in the shorter axis of the primary tumor predicted pathological response and survival. Our results support the use of the RECIST system for evaluating the effect of NAC on the primary lesion and may contribute to more consistent collection of institutional data that could be used for future research.

## Supplementary Information

**Additional file 1: Supplementary Table S1**. Correlation analysis of inter-examiner variability. **Supplementary Table S2**. Logistic regression analysis for estimating pathologically ineffective response (TRG 0–1a) from RECIST shorter axis, divided by original tumor size (shorter axis). **Supplementary Table S3**. Reduction rates of the longest diameter, shorter axis, and their multiplication of the primary tumor for differentiating a pathologically “effective” (TRG 1b–3) response from an “ineffective” (TRG 0–1a) response using receiver operating characteristic curve analysis. (PPTX 46 kb)

**Additional file 2.**

## Data Availability

The dataset supporting the conclusions of this article is included within the additional file 2.

## Abbreviations

CR: Complete response
CT: Computed tomography
DFS: Disease-free survival
ICC: Intraclass correlation coefficient
NAC: Neoadjuvant chemotherapy
OS: Overall survival
PD: Progressive disease
PET: Positron emission tomography
PR: Partial response
RECIST: Response Evaluation Criteria in Solid Tumors
ROC: Receiver operating characteristic
SD: Stable disease
TRG: Tumor regression grade
