# Supplementary material for: Evaluating the effect of Neoadjuvant chemotherapy for esophageal Cancer using the RECIST system with shorter-axis measurements: a retrospective multicenter study
Source: BMC Cancer. 2021 Sep 9;21:1008. doi: 10.1186/s12885-021-08747-y (PMC8428108; doi:10.1186/s12885-021-08747-y)
Supplement: Supplementary file 1 — Additional file 1: Supplementary Table S1. Correlation analysis of inter-examiner variability. Supplementary Table S2. Logistic regression analysis for estimating pathologically ineffective response (TRG 0–1a) from RECIST shorter axis, divided by original tumor size (shorter axis). Supplementary Table S3. Reduction rates of the longest diameter, shorter axis, and their multiplication of the primary tumor for differentiating a pathologically “effective” (TRG 1b–3) response from an “ineffective” (TRG 0–1a) response using receiver operating characteristic curve analysis. (PPTX 46 kb) [file 12885_2021_8747_MOESM1_ESM.pptx]

## Slide 1
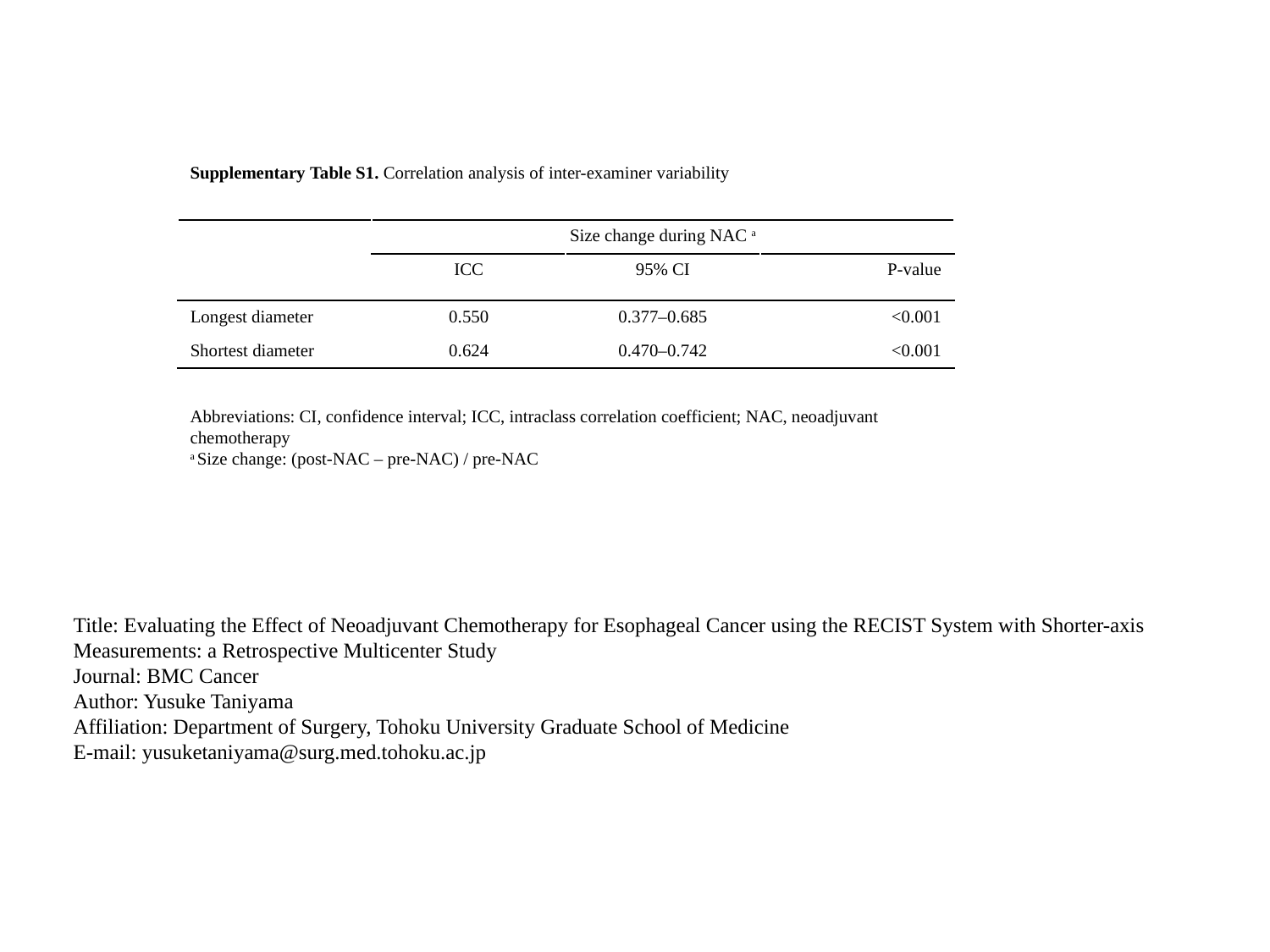

Supplementary Table S1. Correlation analysis of inter-examiner variability
| | Size change during NAC a | | |
| --- | --- | --- | --- |
| | ICC | 95% CI | P-value |
| Longest diameter | 0.550 | 0.377–0.685 | <0.001 |
| Shortest diameter | 0.624 | 0.470–0.742 | <0.001 |
Abbreviations: CI, confidence interval; ICC, intraclass correlation coefficient; NAC, neoadjuvant chemotherapy
a Size change: (post-NAC – pre-NAC) / pre-NAC
Title: Evaluating the Effect of Neoadjuvant Chemotherapy for Esophageal Cancer using the RECIST System with Shorter-axis Measurements: a Retrospective Multicenter Study
Journal: BMC Cancer
Author: Yusuke Taniyama
Affiliation: Department of Surgery, Tohoku University Graduate School of Medicine
E-mail: yusuketaniyama@surg.med.tohoku.ac.jp

## Slide 2
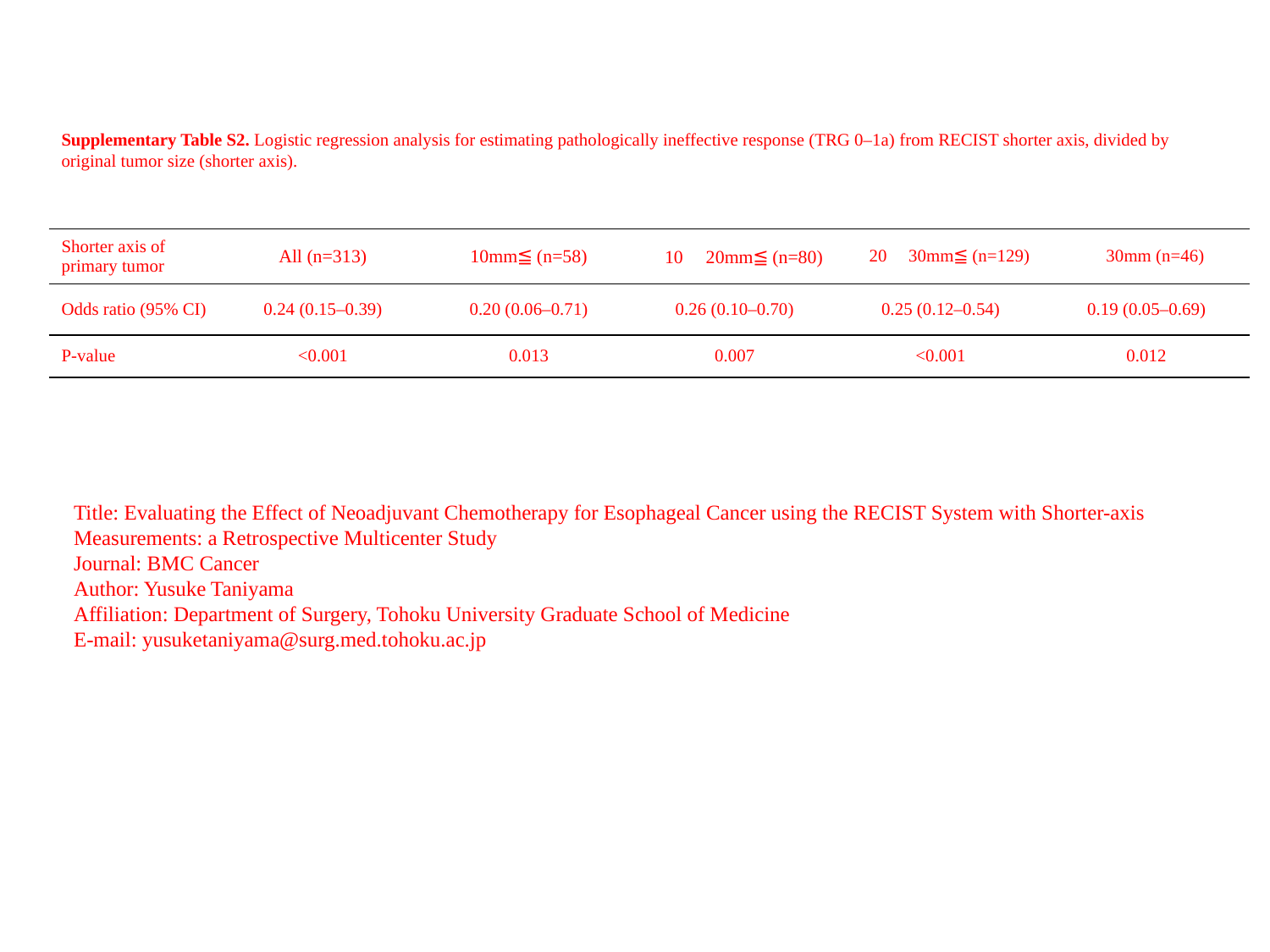

Supplementary Table S2. Logistic regression analysis for estimating pathologically ineffective response (TRG 0–1a) from RECIST shorter axis, divided by original tumor size (shorter axis).
| Shorter axis of primary tumor | All (n=313) | 10mm≦ (n=58) | ＜10～20mm≦ (n=80) | ＜20～30mm≦ (n=129) | ＜30mm (n=46) |
| --- | --- | --- | --- | --- | --- |
| Odds ratio (95% CI) | 0.24 (0.15–0.39) | 0.20 (0.06–0.71) | 0.26 (0.10–0.70) | 0.25 (0.12–0.54) | 0.19 (0.05–0.69) |
| P-value | <0.001 | 0.013 | 0.007 | <0.001 | 0.012 |
| | | | | | |
Title: Evaluating the Effect of Neoadjuvant Chemotherapy for Esophageal Cancer using the RECIST System with Shorter-axis Measurements: a Retrospective Multicenter Study
Journal: BMC Cancer
Author: Yusuke Taniyama
Affiliation: Department of Surgery, Tohoku University Graduate School of Medicine
E-mail: yusuketaniyama@surg.med.tohoku.ac.jp

## Slide 3
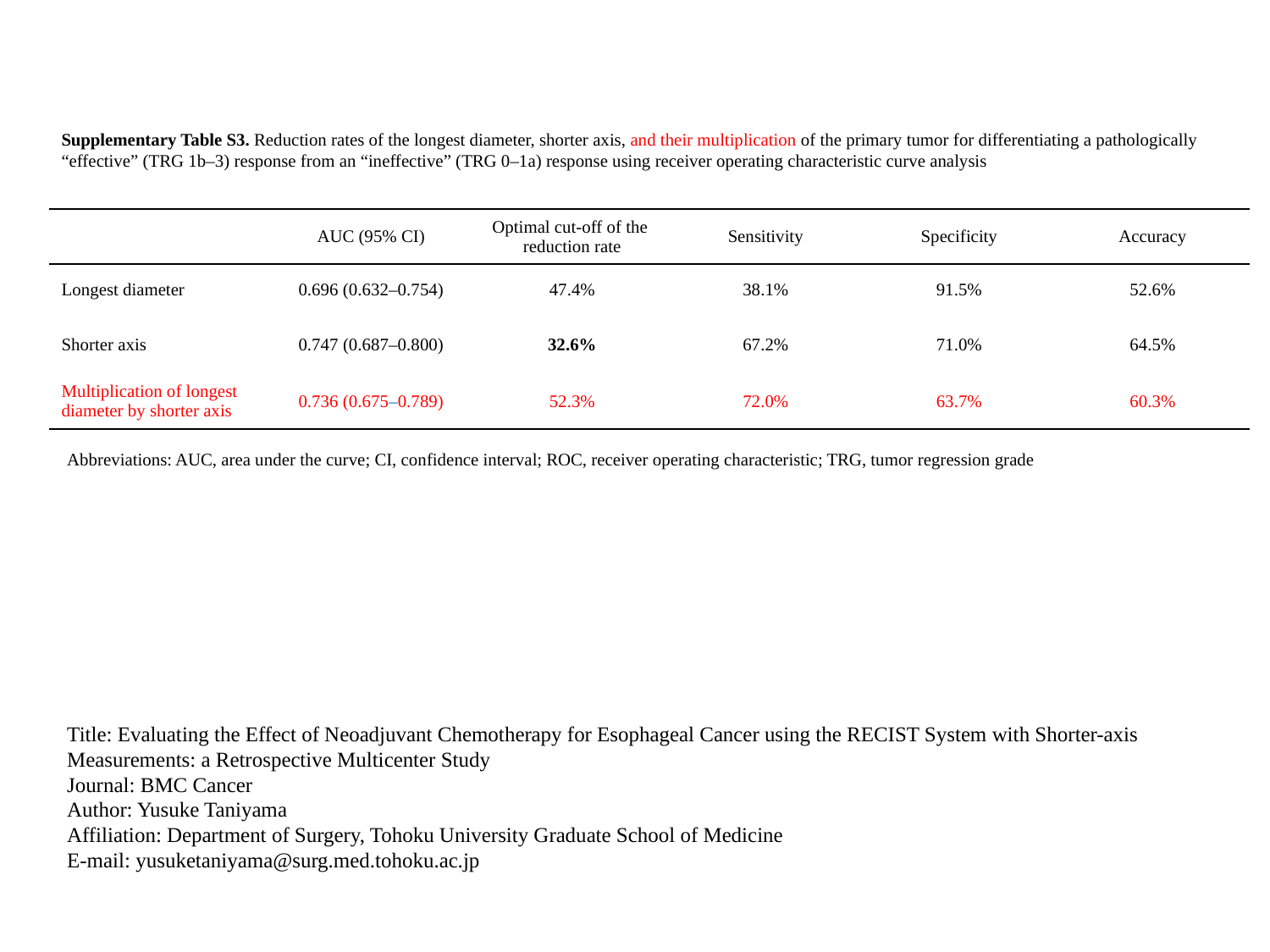

Supplementary Table S3. Reduction rates of the longest diameter, shorter axis, and their multiplication of the primary tumor for differentiating a pathologically “effective” (TRG 1b–3) response from an “ineffective” (TRG 0–1a) response using receiver operating characteristic curve analysis
| | AUC (95% CI) | Optimal cut-off of the reduction rate | Sensitivity | Specificity | Accuracy |
| --- | --- | --- | --- | --- | --- |
| Longest diameter | 0.696 (0.632–0.754) | 47.4% | 38.1% | 91.5% | 52.6% |
| Shorter axis | 0.747 (0.687–0.800) | 32.6% | 67.2% | 71.0% | 64.5% |
| Multiplication of longest diameter by shorter axis | 0.736 (0.675–0.789) | 52.3% | 72.0% | 63.7% | 60.3% |
| | | | | | |
Abbreviations: AUC, area under the curve; CI, confidence interval; ROC, receiver operating characteristic; TRG, tumor regression grade
Title: Evaluating the Effect of Neoadjuvant Chemotherapy for Esophageal Cancer using the RECIST System with Shorter-axis Measurements: a Retrospective Multicenter Study
Journal: BMC Cancer
Author: Yusuke Taniyama
Affiliation: Department of Surgery, Tohoku University Graduate School of Medicine
E-mail: yusuketaniyama@surg.med.tohoku.ac.jp
